# Diagnosis and surgical repair of congenital double aortic arch in infants

**DOI:** 10.1186/s13019-019-0976-x

**Published:** 2019-09-09

**Authors:** Yiting Yang, Xin Jin, Zhengxia Pan, Yonggang Li, Chun Wu

**Affiliations:** Medical master,department of cardiothoracic surgery, Chongqing Medical University Affiliated Children’s Hospital, Chongqing, 400000 People’s Republic of China

**Keywords:** Double aortic arch, Cardiac malformation, Tracheal stenosis, Tracheal stenosis index, Esophagus stenosis, Cardiac surgery

## Abstract

**Objectives:**

Double aortic arch (DAA) is a rare congenital vascular malformation. This study aims to summarize the experience of diagnosis and surgical treatment for congenital double aortic arch.

**Methods:**

The clinical data of 24 cases with double aortic arch (DAA) from January 2008 to January 2018 in our hospital was reviewed retrospectively.

**Results:**

A total of 24 cases, including 12 patients with isolated DAA and 12 patients with DAA and associated intracardiac defects were identified. There were 14 males and 10 females, with an average age of 11 months. The associated intracardiac malformations included ventricular septal defect (VSD), atrial septal defect (ASD), patent ductus arteriosus (PDA), tetralogy of Fallot (TOF), transposition of the great arteries (TGA), pulmonary stenosis (PS), and patent foramen ovale (PFO). Of the 12 patients with DAA and intracardiac malformations, 7 patients underwent intracardiac repair simultaneously, however, 3 patients underwent isolated double aortic arch correction. One patient with DAA and TGA underwent surgical correction of congenital vascular ring at the first stage, and the arterial switch operation was performed at the second stage. The clinical outcomes of 23 patients were promising, however, in one patient, parents decided not to do the surgery due to personal reasons. The average follow-up time was 35 months.

**Conclusions:**

Tracheal and esophageal compression are commonly seen in patients with DAA, however could be relieved significantly after surgery. In particular cases, the simultaneous intracardiac defects repair could be performed. Misdiagnosis was easily established with isolated echocardiography. Fortunately, the correct diagnosis of DAA and associated intracardiac defects could be established with the use of combined chest computed tomography.

## Introduction

The Congenital Double Aortic Arch (DAA) which accounts for 46–76% of the complete rings is the most common vascular malformation in the congenital annulus [1, 2]. Because of the persistence of the fourth aortic arches during embryonic development, both aortic arches are from the ascending aorta, bypassing the trachea and esophagus, inflowing into the descending aorta [3]. The anatomy of the double aortic arch is mainly the right arch sends out the right subclavian artery and the right common carotid artery. The left part of the left arch produces the left subclavian artery and the left common carotid artery. Generally, the right bow dominant type accounts for about 70%,the left arch dominant type accounts for 20,and 5% double bow balanced. The double aortic arch is not only open with both arches but also a bow closure. The right bow is 40% of the left bow, and the left bow is about 20% [4].

Tracheal and esophageal compression are commonly seen in patients with DAA. In the early stage,the symptoms of tracheal compression are more obvious,such as repeated pulmonary infection, wheeze, shortness of breath and so on. When eating liquid food, it is less difficult to swallow due to esophageal stricture. The difficulty of swallowing is relatively obvious as times go on, so early intervention is very important.

## Material and methods

**Fig. 1:**
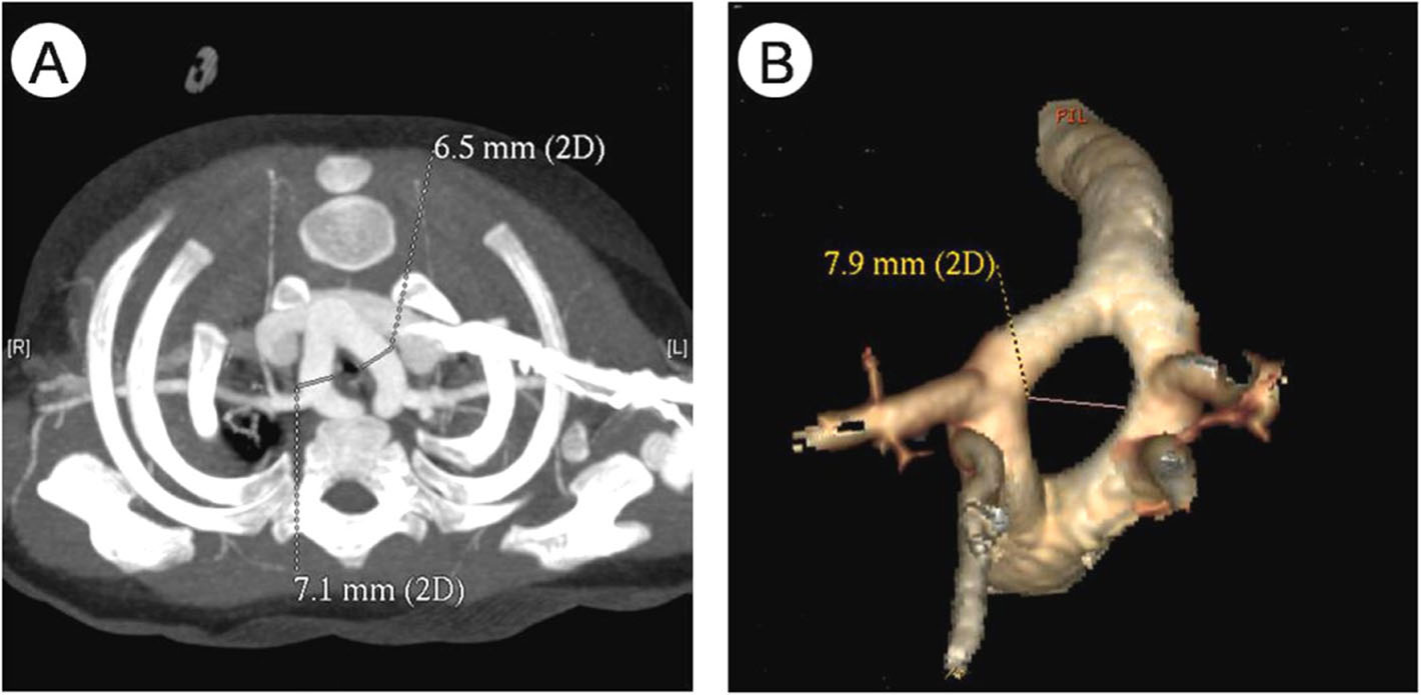
**a**:CT tomography of children with DAA **b**: Three-dimensional reconstruction of vascular rings of children with DAA

During the period between January 2008 and January 2018, 24 patients with DAA were treated at our center, including 14 males and 10 females; aged 29 days to 4 years (11 months 21 days ±13 months 6 days); median age is 8 months 4 days. The body mass is 3.5~20.0 kg (8.13 ± 4.36 kg), and the median is 6.75 kg.The infants who were complained of “cough, wheeze, shortness of breath, and finding heart murmurs”, were misdiagnosed with “pneumonia, bronchitis, wheeze, respiratory failure” and other respiratory diseases. Among them, there were 16 cases of cough, 12 cases of wheeze, 8 cases of shortness of breath, 2 cases of fever, 3 cases of cyanosis, 1 case of heart murmur, and 5 cases of sputum. Fourteen cases were diagnosed as “pneumonia” before surgery. After undergoing echocardiography,chest CT, three-dimensional reconstruction of trachea, all patients were diagnosed with DAA clearly and treated in our department. Among them, 12 cases had other congenital intracardiac malformations, including 7 cases of VSD, 7 cases of ASD, 4 cases of PFO,1 case of TGA,2 cases of TOF,and 6 cases of PDA. Chest CT and three-dimensional reconstruction of airway were performed before operation,and the diameter of the stenosis segment and the length of the stenosis were recorded (Fig. 1). We introduce the concept of tracheal stenosis index, which means DTS/LTS × 100%, can be used as a quantitative indicator to determine the degree of tracheal stenosis, the smaller the index value, the more severe the tracheal stenosis. In this group of patients, the mean diameter of the tracheal stenosis was 3.40 ± 1.08 mm, the average stenosis length was 8.29 ± 2.91 mm, and the average stenosis index was 50.15% ± 39.43%. Preoperative esophageal barium meal examination should be perfected to determine whether esophagus is narrow or not. Esophageal and tracheal stenosis are shown in Table 1.

**Table 1:** preoperative trachea and esophageal stenosis

Trachea \ esophagus	No	T2-T3	T3-T4	T4-T5
No	/	/	/	/
T3	5	4	/	1
T4	1	1	1	/
T4-T5	6	/	2	/
T5-T6	1	1	1	/

Remarks: T2: second thoracic level

Remarks:DTS:(the diameter of tracheal stenosis); LTS: (the length of tracheal stenosis).

### Surgery

In this group, all patients underwent surgery under general anesthesia except one decided not to do the surgery due to personal reasons, 12 cases of simple DAA were performed under normal temperature without cardiopulmonary bypass. The left thoracic posterolateral incision was inserted into the chest, and the secondary arch was fully dissociated. The ductus arteriosus or ligament was ligated and removed, and the compression of the esophageal trachea was relieved. At the secondary arch junction, the blood flow was ensured, and the blood pressure and oxygen saturation of the upper and lower limbs were monitored, and the inferior bow was broken. Twelve patients with intracardiac malformations were treated at the same period with a median transthoracic approach under cardiopulmonary bypass. See Table 2 for details.

**Table 2:** General condition of 12 patients with intracardiac malformation

Case	Intracardiac malformation	Surgery	Length of operation (min)	cardiopulmonary bypass time (min)	Aortic crossclamp time, ACCT (min)	Mechanical ventilation (hr)	PICU(d)	CCU(d)	Length of hospital stay, LOS (d)	Length of postoperative (d)
1	VSD/PDA	Correction of DAA (right arch dissection) + end-to-side anastomosis of the descending aortic and right common carotid artery + VSD repair + PDA ligation under cardiopulmonary bypass	410	252	134	96	9	3	37	20
2	ASD/VSD	Correction of DAA (right arch dissection) + left pulmonary artery patch enlargement + VSD repair + ASD repair under cardiopulmonary bypass	325	107	54	48	7	3.50	46	32
3	ASD/VSD	Correction of DAA (left arch dissection) + PDA ligation + VSD repair + ASD repair under cardiopulmonary bypass	195	84	35	49	8	8	35	15
4	VSD/PDA/PFO	Correction of DAA (left arch dissection) + PDA ligation + VSD repair + PFO repair under cardiopulmonary bypass	90	71	20	117	6	3	27	14
5	TOF/PDA	Correction of DAA (left arch dissection) + total repair of TOF + PDA ligation under cardiopulmonary bypass	345	186	110	47	5	2	32	24
6	TGA/VSD/PS	Correction of DAA (left arch dissection)	70	/	/	22	2	3	12	10
7	ASD/PDA	Correction of DAA (left arch dissection) + PDA ligation	61	/	/	138	2	3	58	36
8	ASD/VSD	Correction of DAA (left arch dissection) + VSD repair + ASD repair under cardiopulmonary bypass	200	98	35	26	3	5	28	17
9	ASD	Correction of DAA (left arch dissection)	85	/	/	/	/	5	17	11
10(no surgery)	ASD/VSD	/	/	/	/	/	/	/	7	
11	TOF/ASD/PDA	Correction of DAA (left arch dissection) + total repair of TOF + PDA ligation under cardiopulmonary bypass	195	119	61	144	8	10	23	18
12	PDA	Correction of DAA (left arch dissection)	70	/	/	24	/	5	18	

Remarks: minute (min), hour (hr),day(d)

### Follow-up and statistical methods

We followed up this group of patients regularly. The chest radiograph, echocardiography and CT airway reconstruction were reviewed according to the situation, and the prognosis of the children was evaluated according to the clinical manifestations. The follow-up time ranged from 1 month to 92 months postoperatively. The data was analyzed by using SPSS 20.0 statistical software. The normal distribution continuity data was described by ^−^X ± S, and the mean comparison between the two samples was performed by t test. *P* < 0.05 was considered statistically significant.

## Results

### Generally results

All the 23 patients were cured and discharged from hospital after the surgery. The follow-up time was 1~92 (35.22 ± 30.26) months after operation. There was no near-long-term death, and the respiratory symptoms were improved significantly compared with preoperative. Tracheal and esophageal development were improved, and there was no significant difference between the same age. One patient who gave up the surgery for own reason was followed up for death, and the reason was unknown. The postoperative ventilator duration was 64.63 ± 50.38 h, the median time was 48.00 h, the average duration of postoperative monitoring was 9.14 ± 4.58 days, and the median time was 8.00 days. In simple aortic arch disconnection, the average duration of postoperative ventilator was 5.88 ± 5.95 h, the median duration was 4.00 h, the average length of postoperative monitoring was 4.00 ± 1.65 days, and the median duration was 4.00 days. The correlation and comparison of different superior arches on tracheal compression are shown in Tables 3 and 4. The results showed no statistically significant differences.

**Table 3:** Correlation between superior arch and tracheal stenosis

Position	Left superior arch	Right superior arch
T3	3	7(1unsurgical)
T4	3	/
T4-T5		8
T5-T6		3

**Table 4:** Comparison of superior arch and tracheal stenosis

Group	case	the diameter of tracheal stenosis (mm)	the length of tracheal stenosis (mm)	tracheal stenosis index(%)
Left superior arch	6	3.30 ± 1.59	8.28 ± 2.35	39.27 ± 12.53
Right superior arch	18	3.43 ± 0.90	8.29 ± 3.14	53.78 ± 44.74
t		−0.149	0.842	−0.942
P		> 0.05(0.887)	> 0.05(0.438)	> 0.05(0.389)

### Postoperative complications and follow-up

Chylothorax, left vocal cord sputum, transient hypertension are the common postoperative complications [5, 6]. In this group of patients,including bilateral lung exudative lesions(*n* = 6), atelectasis(*n* = 1),pneumonia(*n* = 1),pleural effusion(*n* = 2),respiratory failure(*n* = 5), pneumothorax(*n* = 2),and subcutaneous emphysema(*n* = 3),coagulopathy(*n* = 2), chylothorax(*n* = 2). One chylothorax that was cured by low-fat diet, continuous intra-thoracic injection of azithromycin for 3 days and continuous thoracic drainage. The other was treated with lymphatic suture through the original incision on the 18th day after operation and got postoperative improvement. All complications were cured after treatment. All postoperative blood gas analysis of the children showed no carbon dioxide retention. Clinical symptoms were all relieved to varying degrees. Compared with preoperative, postoperative airway reconstruction showed that tracheal and esophageal stenosis were improved significantly. Among them, 18 patients whose mean tracheal stenosis index was 53.98% ± 43.21% and average follow-up time was 35.72 ± 30.96 months had no obvious tracheal stenosis in the three-dimensional reconstruction of the trachea. Five patients were improved significantly compared with preoperative, the average tracheal stenosis index was 29.60% ± 11.12% before operation, while 55.88% ± 19.00% after operation, that the average follow-up time was 33.40 ± 30.97 months. The tracheal stenosis index was significantly higher than that before surgery,which mean the degree of stenosis was reduced significantly. Simultaneously, the clinical symptoms were relieved completely. It can be seen that the compression stenosis is improved significantly during the growth and development of children.

### Typical case

**Fig. 2:**
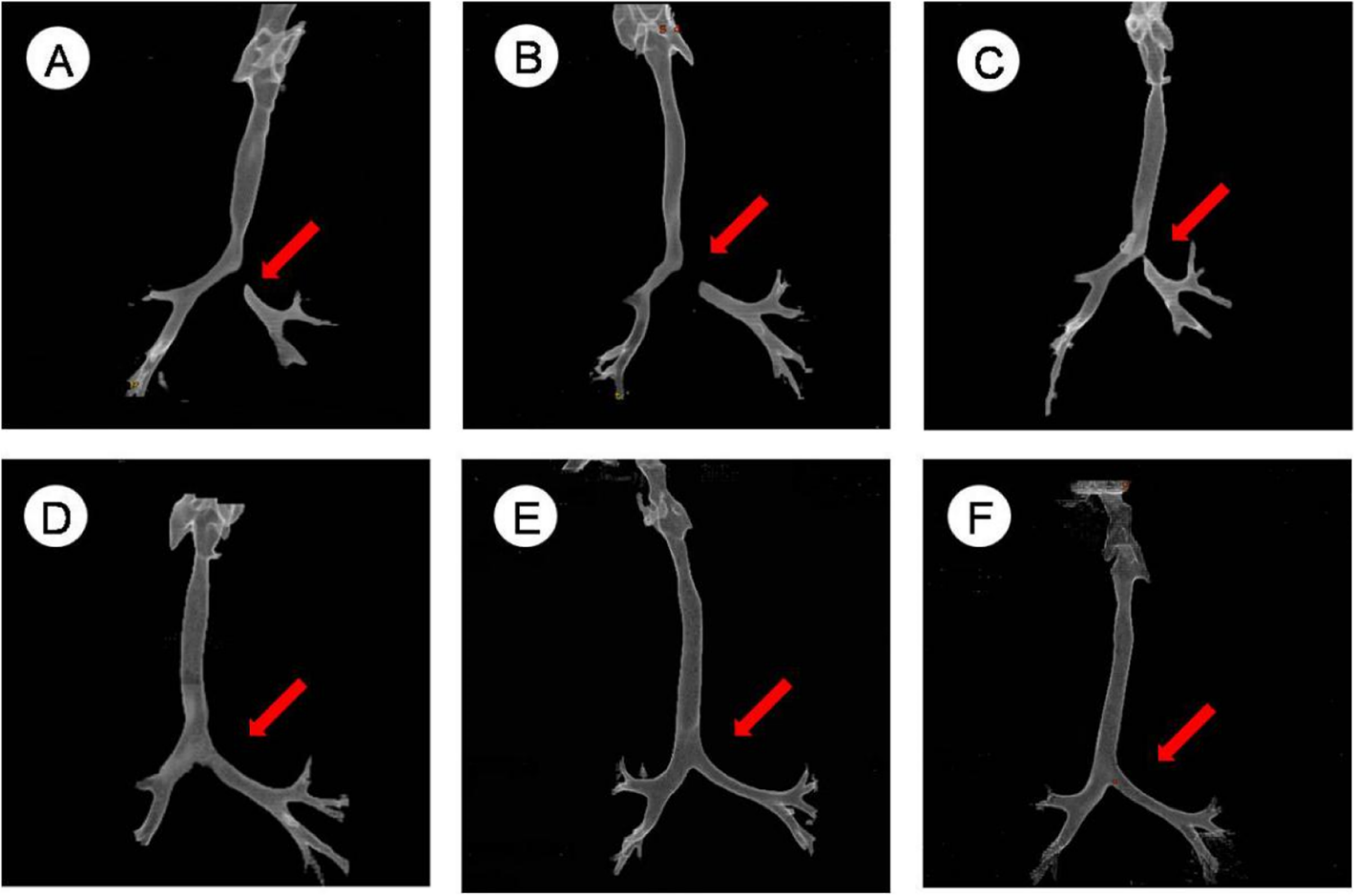
Relationship between follow-up time and tracheal reconstruction **a**:preoperation; **b**:2 months after operation; **c**:8 months after operation; **d**:13 months after operation; **e**:20 months after operation; **f**:51 months after operation

A 3-month-20-day-old female infant was hospitalized in our respiratory medicine because of “pneumonia” on 2014-03-09. After the relevant symptomatic treatment, she still have the lip and complexion cyanosis after crying, licking milk and the other discomforts. After the relevant examinations were completed, the diagnosis of the DAA which was combined with ASD and PDA was clearly. The lower trachea and tracheal bifurcation,the left main bronchial initial segment were compressed,the narrowest diameter was 3.46 mm,the stenosis length was 8.93 mm,the tracheal stenosis index was 38.76%.We did double aortic arch surgery (left arch dissection), arterial catheterization suture in 2014-03-30 under the general anesthesia. During the intraoperative, the width of left and right arch were about 2.5 mm and 6.5 mm, tube type PDA(0.25 cm). The child recovered smoothly after surgery and was discharged. The clinical symptoms were relieved completely during the outpatient follow-up for 51 months, Compared with the same age children, there was no significant difference in tracheal and esophageal development, and no stenosis was seen. Tracheal reconstruction is shown in Fig. 2.

## Discussion

DAA is the most common type of complete vascular ring, it surrounds the trachea and esophagus completely, compresses them and causes different degrees of tracheal stenosis, esophageal obstruction. For patients with intractable wheeze, repeated lung infections, especially those with eating obstruction, the vascular ring may be suspected highly. The etiology of DAA is not clear. It has been reported that its pathogenesis is related to genetic factors and its correlation with chromosomal abnormalities is as high as 24% [7]. McElhinney et al. studied 66 cases of 22q11 chromosome microdeletions, 14% of which had clinical symptoms of DAA, which became an important etiological factor in the congenital double aortic arch [8, 9]. DAA often combines with other cardiovascular malformations, TOF is the most common [10]. The reason may be related to the loss of genes on 22q11 leading to conical septum and macrovascular dysplasia during the embryonic development. However, in this group of cases, intracardiac malformation is more common with ASD and VSD, only 2 cases of TOF, which tells us the disease-related gene deletion needs to be further explored. And the correlation between 22q11 and disease is mentioned in the relevant literature. Due to the limitations of national conditions, there is no relevant chromosome or genetic examination for this group of patients, so we have not further explored it. Unfortunately, We regret it. And in the subsequent research, it must be more rigorous and more careful.

In terms of diagnosing, infant MRI requires general anesthesia or deep sedation with a long time. Therefore, the use of MRI is limited for the infants with vascular rings who usually have respiratory symptoms,including wheeze, dyspnea, respiratory failure, apnea, requiring intubation or mechanical ventilation [11, 12]. Fiberoptic bronchoscopy can observe tracheal stenosis clearly, but it is not necessary because of the risk of stimulating bronchospasm and aggravating airway obstruction. Cardiovascular angiography,as a preoperative examination,is not recommended because it is an invasive procedure. In this group of patients, only 13 patients were diagnosed by ultrasound. Therefore, we should consider the DAA when the left and right aortic arches were detected by echocardiography, and further detected the number of branches of the brachiocephalic artery. If only two brachiocephalic arteries are seen on each side of the arterial arch, the possibility of a double aortic arch is extremely high. But its disadvantage is that it cannot confirm the compression of the surrounding tissue caused by the vascular ring, and can not find the closed arch and arterial ligaments with no blood flowing. CT can show the shape of the aortic arch and the branch of the brachiocephalic artery, as well as the compressed stenosis of the tracheal esophagus [13]. Furthermore, three-dimensional reconstruction can make up for the deficiency of the cross-sectional image on the long axis of the trachea and bronchus, quantify the diameter and area of the trachea and bronchial lumen accurately [14]. It is beneficial to observe the performance of the narrow segment of the lesion from different angles, such as the length,the location,the severity of tracheal stenosis, the upper and lower boundaries of the lesion and so on. It also shows the complications caused by bronchial stenosis,including localized atelectasis, intraductal tissue adhesion and tissue edema. In this group of patients, we use Brilliance iCT(128) which is made from Philips to complete it. During the process of CT scan,we have always focused on the protection of sensitive organs,such as the thyroid,gonads and so on. The cardiac CT and airway reconstruction were performed under natural ventilation without tracheal intubation or mechanical ventilation no matter in the time of preoperative or postoperative. The PEEP value was not involved, so there is no difference in the assessment of the airway. Therefore, this study believes that echocardiography combined with CT is the best auxiliary examination for preoperative diagnosis and evaluation of DAA.However,the 2000’s report of the United Nations Scientific Committee on the Effects of Atomic Radiation (UNSCEAR) and the No. 68 report of US Committee for Radiation Protection (NCRP) both believe that the lifetime incidence of radiation-induced lethal cancer is 2 to 3 times greater than that of adults since children are in a period of vigorous growth and development. CT has radiation damage, and the sensitive of pediatric radiation damage is 2–3 times greater than adults [15]. Wang Qiang et al [16] have shown that the age has a significant effect on the size-specific dose estimation (SSDE) of children’s chest CT while the sex has no value on it. The Lifetime Attribution Risk (LAR) of lung cancer, gastric cancer, liver cancer, thyroid carcinoma, breast cancer and leukemia caused by radiation doses from pediatric chest CT shows that lung cancer and female breast cancer have higher LAR. When a child under 5 years old receives a radiation dose of 100 mGy for CT, the incidence of fatal cancer increases by about 1% [17]. For this group of patients,it’s necessary to do the CT scan to evaluate the airway,and the radiation doses conform the national regulations. The most important is that we do everything what we can do to protect the sensitive organs to induce the radiation damage.

In terms of treatment, some scholars believe that all patients with vascular rings, even those who are asymptomatic currently, will have obvious airway symptoms in the future as the disease progresses. Early appropriate surgery can avoid serious complications caused by hypoxia and dysphagia. Studies have shown that delayed treatment can cause sudden death and further tracheobronchial injury [18]. The patients in this group were treated surgically after the diagnosis was confirmed, and the effect was good. The combined intracardiac malformation could be cured at the same time, the follow-up effect was good in the near and medium term. However, there are also reports that some DAA patients with balanced development of double arches were found accidentally,and do not have any clinical symptoms. The diameter of vascular ring is approximately the same as that of esophagus and trachea, resulting that the esophagus and trachea were not compressed. No further relevant surgical treatment was performed, and the follow-up was no special [19]. It can be seen that the clinical asymptomatic balanced double aortic arch can be not operated temporarily, long-term follow-up of echocardiography, CT and airway reconstruction to monitor and evaluate the development of double arch, trachea and esophagus is effectively. The purpose of the treatment of the DAA is to disconnect the inferior arch, retain the superior arch, and relieve the compression of the trachea and esophagus. However, there are still different opinions on whether to treat the trachea stenosis and tracheomalacia during the operation. Some scholars believe that the tracheomalacia is not easy to relieve before the age of 2 years. For the severe, diffuse, “O” type tracheal rings, the excision of tracheal stenosis should be performed intraoperatively, end-to-end anastomosis or Slide tracheoplasty, H buckle J [20, 21]. However, in the literature reviewing of DAA, no cases of tracheal stenosis were treated concurrently. Combined with the data of this group, the degree of DAA on the trachea and esophageal compression was relatively light compared with other vascular rings (such as pulmonary artery slings), and most of them were short segments. It could catch up with the normal and no serious complications occurred once the pressure is relieved. Some scholars have found that the tracheal stenosis segment can grow with the age of the patients after relieving the compression, and the tracheal diameter of the stenosis can approach the normal level in most children around 9 years old [21]. At the same time, the data from Toronto Children’s Hospital in Canada showed that conservative treatment of partial stage stenosis is a safe and effective measure. Follow-up showed that the diameter of tracheal stenosis segment before one-year-old was significantly different from that of normal children, and the development was delayed. However, the tracheal development showed a “speed up” phenomenon after that age. It has reached the level of normal children completely at 9-year-old [22]. In this group of patients,there was no significant statistical difference in tracheal compression segment, the tracheal degree of stenosis and the results of long-term follow-up in the comparison of different superior arches (*P* > 0.05). Among the 23 patients who underwent surgical treatment, there was no long-segment tracheal stenosis or tracheomalacia which need to treat at the same time. The clinical manifestations of the children were better than before significantly through follow-up visit. CT (airway reconstruction) scan showed that the related tracheal stenosis was improved to a variety of degrees with the release of vascular ring compression and the growth and development of the children themselves. Cough, wheeze and other respiratory symptoms were improved apprently, and their growth and development are the same as those of normal children of the same age.

In terms of postoperative management, considering the tracheal compression is relieved obviously and the tracheal stenosis is improved to different degrees,the postoperative airway management is the key to rehabilitation. We advocate reducing the damage and stimulation of the tracheal mucosa through continuous positive pressure mechanical ventilation,removing the tracheal intubation as soon as possible and avoiding ventilator-associated pneumonia caused by prolonged mechanical ventilation at the same time. If the airway symptoms are obvious after removal of the tracheal intubation, non-invasive continuous positive pressure ventilation or high-flow humidification oxygen therapy should be applied actively. Simultaneously, strengthening the lung treatments can help the patients to pass the postoperative airway edema peak period successfully [23].

## Conclusion

In summary, DAA is a congenital vascular malformation which is rare and misdiagnosed easily. Echocardiography combined with CT and airway reconstruction can diagnose effectively. Once diagnosed is confirmed, Whether the compression on the trachea must exist, and whether surgical intervention is required,it is controversial. However, as for the patients who are experiencing chronic wheeze, shortness of breath, repeated cough, dyspnea, or (and) dysphagia, especially in poor medical treatment, it is correct for diagnosis and surgical treatment timely. Because the degree of the tracheal stenosis which was caused by DAA is lighter than that of other vascular rings (such as pulmonary sling), it can be temporarily treated without concurrent surgery, and it is possible to return to normal with its own growth and development. The correlation between DAA and 22q11 chromosome microdeletion needs further research, whether it is guided jointly by gene defects, environmental factors, drug induction and other factors. It needs further study on how to prevent the fetus from suffering from DAA effectively during pregnancy, Whether it is possible to diagnose and operate the fetus during fetal development through intrauterine operation to reduce the probability of illness.

## Data Availability

The data and materials in the manuscript are available, and the original data for the relevant results is owned by myself and can be contacted if it’s needed.

## Abbreviations

ASD: Atrial septal defect
CT: Computed tomography
d: Day
DAA: Double aortic arch
DTS: The diameter of tracheal stenosis
hr.: Hour
LTS: The length of tracheal stenosis
min: Minute
PDA: Patent ductus arteriosus
PFO: Patent foramen ovale
PS: Pulmonary stenosis
TGA: Transposition of the great arteries
TOF: Tetralogy of Fallot
VSD: Ventricular septal defect
